# The genome sequence of the Hungarian meadow viper, *Vipera ursinii rakosiensis *Méhely, 1893

**DOI:** 10.12688/wellcomeopenres.22694.1

**Published:** 2024-07-26

**Authors:** Bálint Halpern, Judit Vörös, Ann M. Mc Cartney, Giulio Formenti, Alice Mouton, Wellcome Sanger Institute Tree of Life Management, Samples and Laboratory team, Wellcome Sanger Institute Scientific Operations: Sequencing Operations, Wellcome Sanger Institute Tree of Life Core Informatics team, Tree of Life Core Informatics collective

**Affiliations:** 1 MME Birdlife Hungary, Budapest, Hungary; 2 Department of Systematic Zoology and Ecology, Institute of Biology, ELTE-Eötvös Loránd University, Budapest, Hungary; 3 HUN-REN – ELTE - MTM Integrative Ecology Research Group, Budapest, Hungary; 4 Department of Zoology, Hungarian Natural History Museum, Budapest, Hungary; 5 University of California Santa Cruz, Santa Cruz, California, USA; 6 The Vertebrate Genome Laboratory, The Rockefeller University, New York, New York, USA; 7 SEED Arlon Campus, University of Liège, Arlon, Belgium

**Keywords:** Vipera ursinii rakosiensis, Hungarian meadow viper, genome sequence, chromosomal, Squamata

## Abstract

We present a genome assembly from an individual female *Vipera ursinii rakosiensis* (the Hungarian meadow viper; Chordata; Lepidosauria; Squamata; Viperidae). The genome sequence is 1,625.0 megabases in span. Most of the assembly is scaffolded into 19 chromosomal pseudomolecules, including the W and Z sex chromosomes. The mitochondrial genome has also been assembled and is 17.38 kilobases in length.

## Species taxonomy

Eukaryota; Opisthokonta; Metazoa; Eumetazoa; Bilateria; Deuterostomia; Chordata; Craniata; Vertebrata; Gnathostomata; Teleostomi; Euteleostomi; Sarcopterygii; Dipnotetrapodomorpha; Tetrapoda; Amniota; Sauropsida; Sauria; Lepidosauria; Squamata; Bifurcata; Unidentata; Episquamata; Toxicofera; Serpentes; Colubroidea; Viperidae; Viperinae; *Vipera*; *Vipera ursinii* (Bonaparte, 1835) (NCBI:txid103942); *Vipera ursinii rakosiensis* Méhely 1893 (NCBI:txid394585).

## Background

The Hungarian meadow viper ( *Vipera ursinii rakosiensis*) – a member of the family *Viperidae* – is a steppe form of the *Vipera ursinii* species group ( Ferchaud *et al.*, 2012; Freitas *et al.*, 2020; Nilson & Andrén, 2001; Vörös *et al.*, 2022; Zinenko *et al.*, 2015). This subspecies was originally described by the prominent Hungarian zoologist Lajos Méhely in 1893, type specimens originating from meadows on the banks of the Rákos River, within current boundaries of Budapest ( Méhely, 1894).

The total length of this viper is up to 60 cm, typically with dark dorsal zig-zag pattern on grey or yellowish-brown basal colour, while the white throat gradually gets darker towards the belly, with off-white spots. Sexual dimorphism is less prominent than in other species of the family: males and females are distinguishable by total and tail length ratio and subcaudal scale numbers.

Distribution of the Hungarian meadow viper is restricted to the Carpathian-Basin. Only fragmented populations survived in the Hanság and Kiskunság regions of Hungary and Transylvanian region of Romania, while it went extinct in Austria due to cultivation changes destroying its prime habitats. This snake is an inhabitant of steppe remnants. Meadows and pastures that form a mosaic of wet and dry grass habitats is favoured by the species, providing the preferred high microclimatic diversity and abundance of prey ( Korsós *et al.*, 2000).

The Hungarian meadow viper is mainly insectivorous, consuming grasshoppers, crickets and small-sized lizards. Adults opportunistically predate on vertebrates such as lizards and rodents. They are diurnal, tending to start each morning basking, usually avoiding being exposed too much as they are predated by many other species.

From October to March, snakes spend the winter underground in abandoned burrows. Males emerge from hibernation in the middle of March, 2 to 4 weeks before females. After a few weeks they start to shed their skin, then begin searching for mates. Females shed their skins after the mating season is over. Hungarian meadow vipers are ovoviviparous, females giving birth to average 10 live offspring usually in the end of July or beginning of August. Newborn snakes shed their skin immediately after they leave the transparent sac in which they were born, then they start their individual life, which is filled with challenges ( Halpern, 2007; Korsós *et al.*, 2000).

The Hungarian meadow viper is Europe’s most endangered venomous snake. It was declared protected in Hungary in 1974 and it is strictly protected since 1988. The subspecies is included in the Bern Convention Appendix II [Council of Europe, 1979 (revised 2002)], and is listed in Appendix I of the Convention on International Trade in Endangered Species of Wild Flora and Fauna ( CITES, 1987) and listed on the International Union for Conservation of Nature (IUCN) Red List as Endangered ( IUCN, 1996) and also listed on the annex II of the Habitats Directive (Council Directive 92/43/EEC 1992), meaning that its occurrences have to be included in the Natura2000 Network of protected areas ( Edgar & Bird, 2005).

As the severe decline of the subspecies was noted by experts by late 1990s, a cooperative conservation effort was initiated in Hungary to save the species from extinction. The Species Conservation Plan targeted to restore populations and habitats with various measures, including captive breeding and reintroduction to reconstructed habitats ( Dankovics *et al.*, 2004; Halpern, 2007). The ex-situ populations’ genetic screening was a necessity from the very beginning of the programme ( Péchy *et al.*, 2015; Vörös *et al.*, 2022), raising various taxonomy or conservation related questions, in which the availability of a precise reference genome opens new horizons. The available reference sequence can also help recent and future efforts, working on more detailed analysis of the phylogeny and phylogeography of the *Vipera ursinii* species-complex, using next generation sequencing methods.

### Genome sequence report

**Figure 1.**
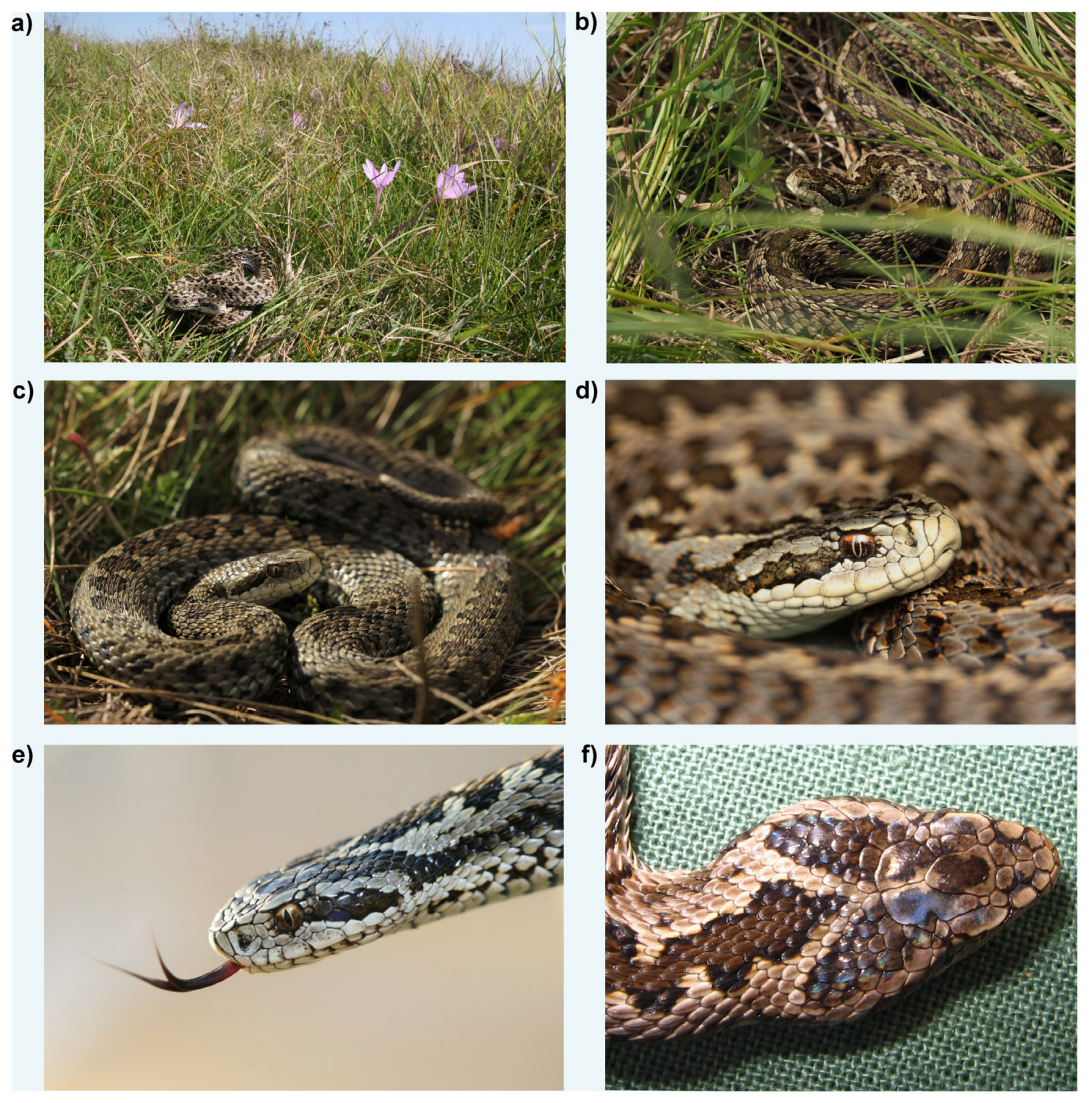
Photographs of *Vipera ursinii rakosiensis:* ( **a**) basking female *in situ* in autumnal setting in Kiskunság ( **b**) female concealed in dry grass as a defence against predators; ( **c**) individual basking exposed; ( **d**– **e**) characteristically, this species has vertical pupils and predominantly pale, unmarked labial scales; ( **f**) variability in head scale arrangement in Hungarian meadow vipers is substantial, enabling individual identification in conservation efforts – shown is specimen rVipUrs1, utilised for genome sequencing.

The genome was sequenced from one female *Vipera ursinii rakosiensis* ( Figure 1) collected from Budapest Zoo, Budapest, Hungary (47.15, 19.31). A total of 28-fold coverage in Pacific Biosciences single-molecule HiFi long reads was generated. Primary assembly contigs were scaffolded with chromosome conformation Hi-C data. Manual assembly curation corrected 204 missing joins or mis-joins and removed 3 haplotypic duplications, reducing the scaffold number by 21.63%, and increasing the scaffold N50 by 0.52%.

**Figure 2.**
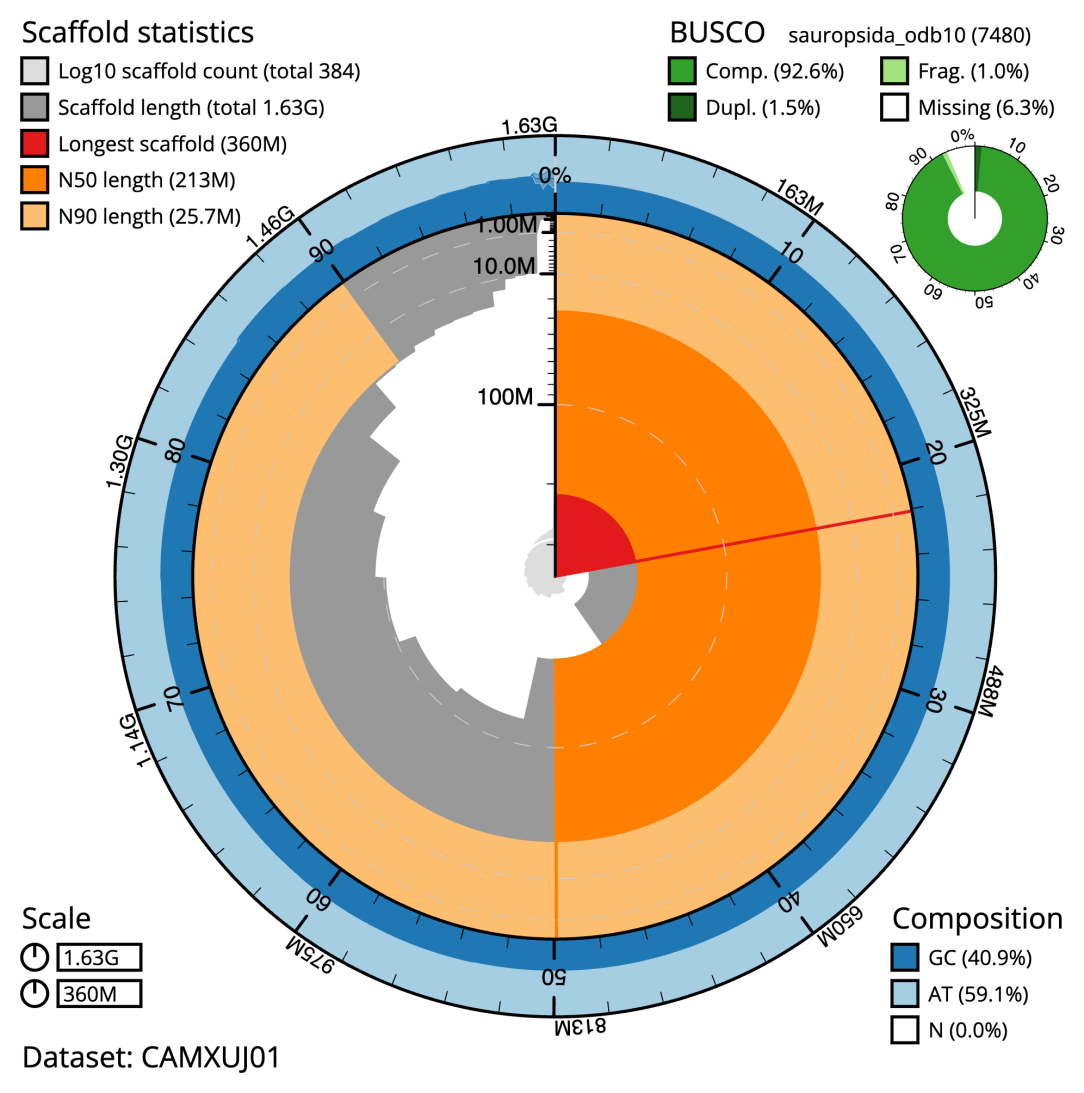
Genome assembly of *Vipera ursinii rakosiensis*, rVipUrs1.1: metrics. The BlobToolKit snail plot shows N50 metrics and BUSCO gene completeness. The main plot is divided into 1,000 size-ordered bins around the circumference with each bin representing 0.1% of the 1,625,023,540 bp assembly. The distribution of scaffold lengths is shown in dark grey with the plot radius scaled to the longest scaffold present in the assembly (359,753,992 bp, shown in red). Orange and pale-orange arcs show the N50 and N90 scaffold lengths (212,821,320 and 25,739,966 bp), respectively. The pale grey spiral shows the cumulative scaffold count on a log scale with white scale lines showing successive orders of magnitude. The blue and pale-blue area around the outside of the plot shows the distribution of GC, AT and N percentages in the same bins as the inner plot. A summary of complete, fragmented, duplicated and missing BUSCO genes in the sauropsida_odb10 set is shown in the top right. An interactive version of this figure is available at https://blobtoolkit.genomehubs.org/view/CAMXUJ01/dataset/CAMXUJ01/snail.

**Figure 3.**
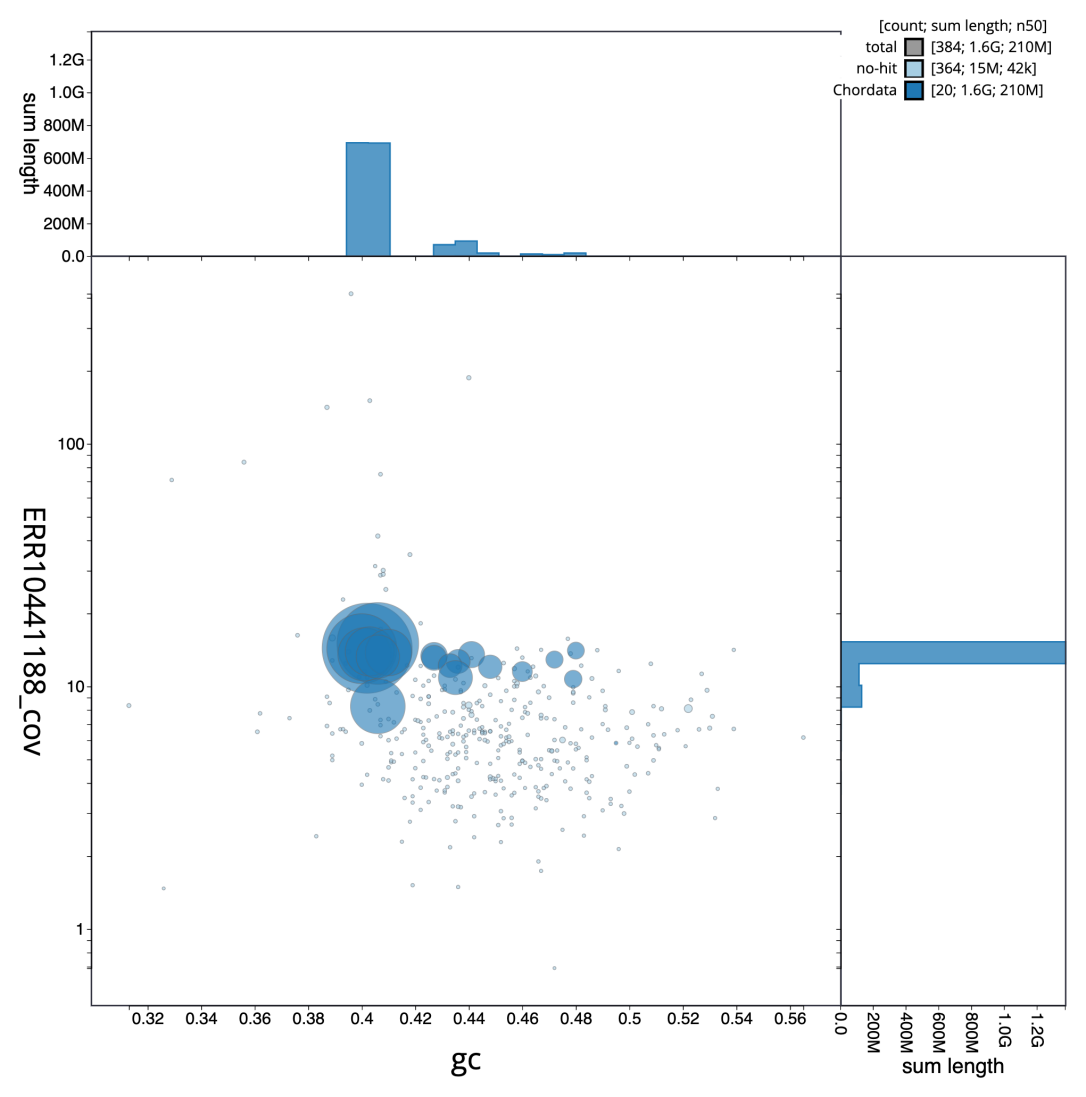
Genome assembly of *Vipera ursinii rakosiensis*, rVipUrs1.1: BlobToolKit GC-coverage plot. Sequences are coloured by phylum. Circles are sized in proportion to sequence length. Histograms show the distribution of sequence length sum along each axis. An interactive version of this figure is available at https://blobtoolkit.genomehubs.org/view/CAMXUJ01/dataset/CAMXUJ01/blob.

**Figure 4.**
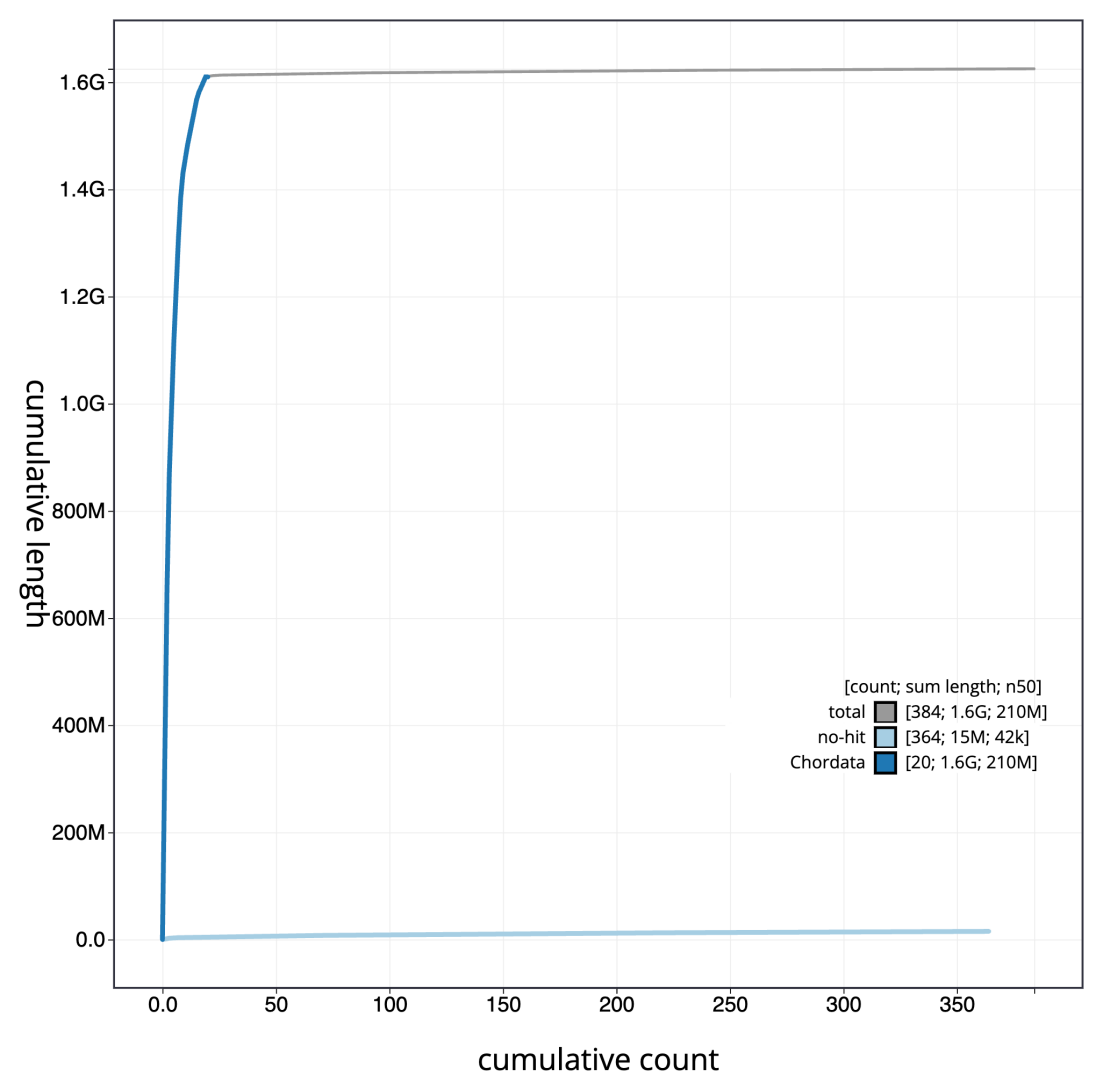
Genome assembly of *Vipera ursinii rakosiensis*, rVipUrs1.1: BlobToolKit cumulative sequence plot. The grey line shows cumulative length for all sequences. Coloured lines show cumulative lengths of sequences assigned to each phylum using the buscogenes taxrule. An interactive version of this figure is available at https://blobtoolkit.genomehubs.org/view/CAMXUJ01/dataset/CAMXUJ01/cumulative.

**Figure 5.**
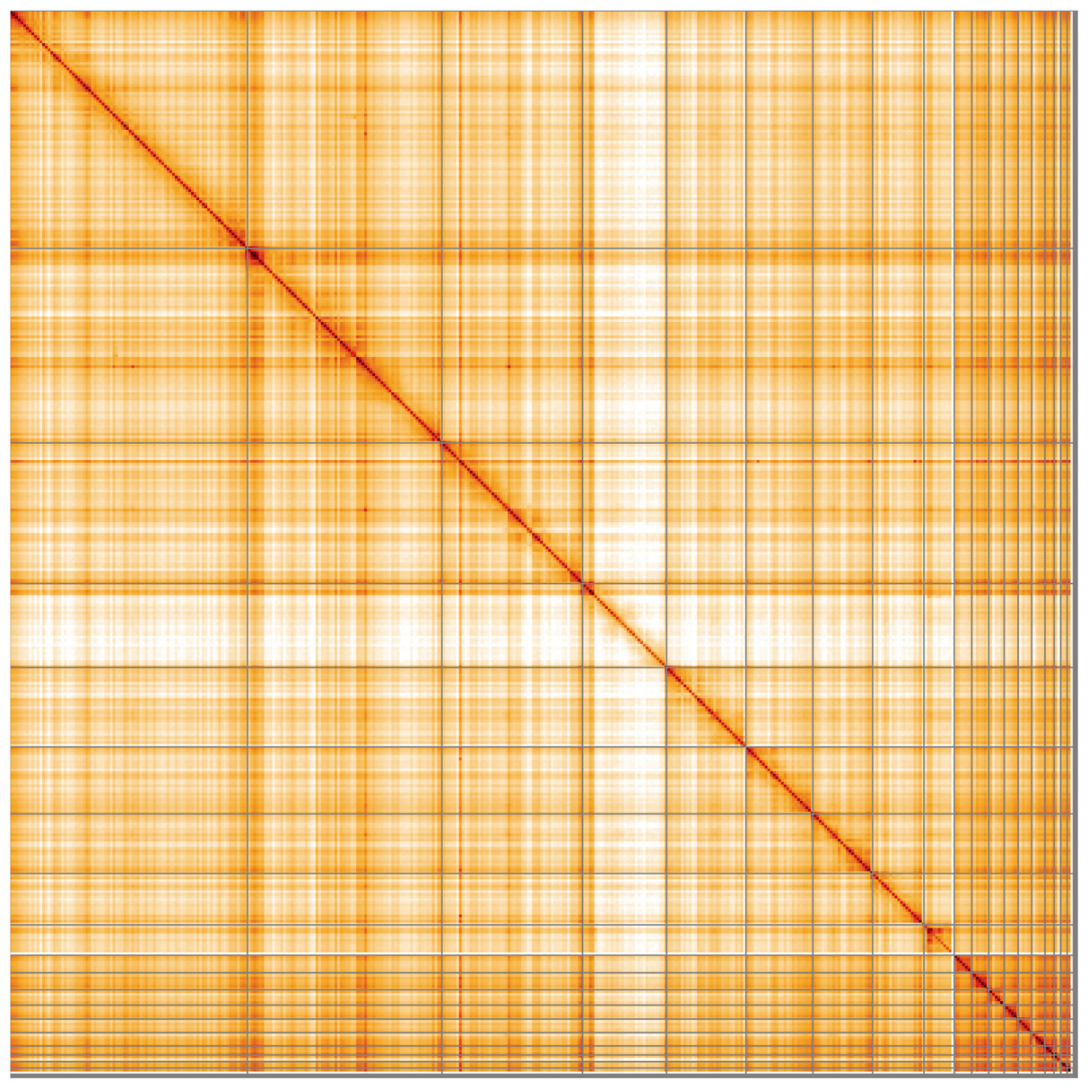
Genome assembly of *Vipera ursinii rakosiensis*, rVipUrs1.1: Hi-C contact map of the rVipUrs1.1 assembly, visualised using HiGlass. Chromosomes are shown in order of size from left to right and top to bottom. An interactive version of this figure may be viewed at https://genome-note-higlass.tol.sanger.ac.uk/l/?d=JtI56nHeRdeUI_b0Pv8Kow.

The final assembly has a total length of 1,625.0 Mb in 383 sequence scaffolds with a scaffold N50 of 212.8 Mb ( Table 1). The snail plot in Figure 2 provides a summary of the assembly statistics, while the distribution of assembly scaffolds on GC proportion and coverage is shown in Figure 3. The cumulative assembly plot in Figure 4 shows curves for subsets of scaffolds assigned to different phyla. Most (99.31%) of the assembly sequence was assigned to 19 chromosomal-level scaffolds, representing 17 autosomes and the W and Z sex chromosomes. Chromosome-scale scaffolds confirmed by the Hi-C data are named in order of size ( Figure 5; Table 2). The order and orientation of chromosome W is not determined with full certainty. While not fully phased, the assembly deposited is of one haplotype. Contigs corresponding to the second haplotype have also been deposited. The mitochondrial genome was also assembled and can be found as a contig within the multifasta file of the genome submission.

**Table 1.** Genome data for *Vipera ursinii rakosiensis*, rVipUrs1.1.

Project accession data		
Assembly identifier	rVipUrs1.1	
Species	*Vipera ursinii rakosiensis*	
Specimen	rVipUrs1	
NCBI taxonomy ID	103942	
BioProject	PRJEB55895	
BioSample ID	SAMEA12832258	
Isolate information	rVipUrs1, female: blood sample (PacBio, Hi-C and RNA sequencing)	
Assembly metrics *		*Benchmark*
Consensus quality (QV)	57.3	*≥ 50*
*k*-mer completeness	99.99%	*≥ 95%*
BUSCO **	C:92.6%[S:91.1%,D:1.5%], F:1.0%,M:6.3%,n:7,480	*C ≥ 95%*
Percentage of assembly mapped to chromosomes	99.31%	*≥ 95%*
Sex chromosomes	WZ	*localised homologous pairs*
Organelles	Mitochondrial genome: 17.38 kb	*complete single alleles*
Raw data accessions		
PacificBiosciences SEQUEL II	ERR10441188, ERR10441187	
Hi-C Illumina	ERR10177764	
PolyA RNA-Seq Illumina	ERR10908602	
Genome assembly		
Assembly accession	GCA_947247035.1	
*Accession of alternate haplotype*	GCA_947247025.1	
Span (Mb)	1,625.0	
Number of contigs	2,203	
Contig N50 length (Mb)	2.1	
Number of scaffolds	383	
Scaffold N50 length (Mb)	212.8	
Longest scaffold (Mb)	359.84	

* Assembly metric benchmarks are adapted from column VGP-2020 of “Table 1: Proposed standards and metrics for defining genome assembly quality” from Rhie *et al.* (2021).

** BUSCO scores based on the sauropsida_odb10 BUSCO set using version 5.3.2. C = complete [S = single copy, D = duplicated], F = fragmented, M = missing, n = number of orthologues in comparison. A full set of BUSCO scores is available at https://blobtoolkit.genomehubs.org/view/CAMXUJ01/dataset/CAMXUJ01/busco.

The estimated Quality Value (QV) of the final assembly is 57.3 with *k*-mer completeness of 99.99%, and the assembly has a BUSCO v5.3.2 completeness of 92.6% (single = 91.1%, duplicated = 1.5%), using the sauropsida_odb10 reference set ( *n* = 7,480).

Metadata for specimens, barcode results, spectra estimates, sequencing runs, contaminants and pre-curation assembly statistics are given at https://links.tol.sanger.ac.uk/species/103942.

**Table 2.** Chromosomal pseudomolecules in the genome assembly of *Vipera ursinii rakosiensis*, rVipUrs1.

INSDC accession	Chromosome	Length (Mb)	GC%
OX365964.1	1	359.75	40.0
OX365965.1	2	294.45	40.5
OX365966.1	3	212.82	40.0
OX365968.1	4	120.14	40.0
OX365969.1	5	101.37	40.5
OX365970.1	6	90.32	41.0
OX365971.1	7	77.67	40.5
OX365973.1	8	26.54	42.5
OX365974.1	9	25.74	44.0
OX365975.1	10	23.23	42.5
OX365976.1	11	21.06	43.5
OX365977.1	12	20.68	43.5
OX365978.1	13	19.98	45.0
OX365979.1	14	13.74	46.0
OX365980.1	15	10.18	48.0
OX365981.1	16	9.66	47.0
OX365982.1	17	9.35	48.0
OX365972.1	W	45.96	43.5
OX365967.1	Z	127.14	40.5
OX365983.1	MT	0.02	41.0

## Methods

### Sample acquisition and nucleic acid extraction

Blood sample was collected from an adult female individual (2-gy-02/09, specimen ID ERGA_BP_HU_01, ToLID rVipUrs1), originating from Dabas population in Central Hungary. The female is a founder of the ex-situ captive population in the Hungarian Meadow Viper Conservation Centre. The blood sampling took place in the Clinic of Budapest Zoo, drawing blood from the caudal vein of the live animal. The sample was placed in liquid nitrogen and later transferred to the Laboratory of Molecular Taxonomy of the Hungarian Natural History Museum where it was stored at –80 °C until cold chain shipping in dry ice to Wellcome Sanger Institute for sequencing.

The workflow for high molecular weight (HMW) DNA extraction at the Wellcome Sanger Institute (WSI) includes a sequence of core procedures: sample preparation; sample homogenisation, DNA extraction, fragmentation, and clean-up. In sample preparation, the rVipUrs1 blood sample was weighed and dissected on dry ice ( Jay *et al.*, 2023). For sample homogenisation, the blood was cryogenically disrupted using the Covaris cryoPREP ^®^ Automated Dry Pulverizer ( Narváez-Gómez *et al.*, 2023).

HMW DNA was extracted using the Automated MagAttract v1 protocol ( Sheerin *et al.*, 2023). DNA was sheared into an average fragment size of 12–20 kb in a Megaruptor 3 system with speed setting 30 ( Todorovic *et al.*, 2023). Sheared DNA was purified by solid-phase reversible immobilisation ( Strickland *et al.*, 2023): in brief, the method employs a 1.8X ratio of AMPure PB beads to sample to eliminate shorter fragments and concentrate the DNA. The concentration of the sheared and purified DNA was assessed using a Nanodrop spectrophotometer and Qubit Fluorometer and Qubit dsDNA High Sensitivity Assay kit. Fragment size distribution was evaluated by running the sample on the FemtoPulse system.

RNA was extracted from an aliquot of the rVipUrs1 blood sample in the Tree of Life Laboratory at the WSI using the RNA Extraction: Automated MagMax™ *mir*Vana protocol ( do Amaral *et al.*, 2023). The RNA concentration was assessed using a Nanodrop spectrophotometer and a Qubit Fluorometer using the Qubit RNA Broad-Range Assay kit. Analysis of the integrity of the RNA was done using the Agilent RNA 6000 Pico Kit and Eukaryotic Total RNA assay.

Protocols developed by the WSI Tree of Life laboratory are publicly available on protocols.io ( Denton *et al.*, 2023).

### Sequencing

Pacific Biosciences HiFi circular consensus DNA sequencing libraries were constructed according to the manufacturers’ instructions. Poly(A) RNA-Seq libraries were constructed using the NEB Ultra II RNA Library Prep kit. DNA and RNA sequencing was performed by the Scientific Operations core at the WSI on Pacific Biosciences SEQUEL II (HiFi) and Illumina NovaSeq 6000 (RNA-Seq) instruments. Hi-C data were also generated from the rVipUrs1 sample, using the Arima2 kit and sequenced on the Illumina NovaSeq 6000 instrument.

### Genome assembly, curation and evaluation

Assembly was carried out with Hifiasm ( Cheng *et al.*, 2021) and haplotypic duplication was identified and removed with purge_dups ( Guan *et al.*, 2020). The assembly was then scaffolded with Hi-C data ( Rao *et al.*, 2014) using YaHS ( Zhou *et al.*, 2023). The assembly was checked for contamination and corrected as described previously ( Howe *et al.*, 2021). Manual curation was performed using HiGlass ( Kerpedjiev *et al.*, 2018) and PretextView ( Harry, 2022). The mitochondrial genome was assembled using MitoHiFi ( Uliano-Silva *et al.*, 2023), which runs MitoFinder ( Allio *et al.*, 2020) or MITOS ( Bernt *et al.*, 2013) and uses these annotations to select the final mitochondrial contig and to ensure the general quality of the sequence.

A Hi-C map for the final assembly was produced using bwa-mem2 ( Vasimuddin *et al.*, 2019) in the Cooler file format ( Abdennur & Mirny, 2020). To assess the assembly metrics, the *k*-mer completeness and QV consensus quality values were calculated in Merqury ( Rhie *et al.*, 2020). This work was done using Nextflow ( Di Tommaso *et al.*, 2017) DSL2 pipelines “sanger-tol/readmapping” ( Surana *et al.*, 2023a) and “sanger-tol/genomenote” ( Surana *et al.*, 2023b). The genome was analysed within the BlobToolKit environment ( Challis *et al.*, 2020) and BUSCO scores ( Manni *et al.*, 2021; Simão *et al.*, 2015) were calculated.

Table 3 contains a list of relevant software tool versions and sources.

**Table 3.** Software tools: versions and sources.

Software tool	Version	Source
BlobToolKit	4.2.1	https://github.com/blobtoolkit/blobtoolkit
BUSCO	5.3.2	https://gitlab.com/ezlab/busco
Hifiasm	0.16.1-r375	https://github.com/chhylp123/hifiasm
HiGlass	1.11.6	https://github.com/higlass/higlass
Merqury	MerquryFK	https://github.com/thegenemyers/MERQURY.FK
MitoHiFi	2	https://github.com/marcelauliano/MitoHiFi
PretextView	0.2	https://github.com/wtsi-hpag/PretextView
purge_dups	1.2.3	https://github.com/dfguan/purge_dups
sanger-tol/ genomenote	v1.0	https://github.com/sanger-tol/genomenote
sanger-tol/ readmapping	1.1.0	https://github.com/sanger-tol/readmapping/tree/1.1.0
YaHS	yahs- 1.1.91eebc2	https://github.com/c-zhou/yahs

### Wellcome Sanger Institute – Legal and Governance

The materials that have contributed to this genome note have been supplied by a Tree of Life collaborator. The Wellcome Sanger Institute employs a process whereby due diligence is carried out proportionate to the nature of the materials themselves, and the circumstances under which they have been/are to be collected and provided for use. The purpose of this is to address and mitigate any potential legal and/or ethical implications of receipt and use of the materials as part of the research project, and to ensure that in doing so we align with best practice wherever possible.

The overarching areas of consideration are:

- Ethical review of provenance and sourcing of the material
- Legality of collection, transfer and use (national and international)

Each transfer of samples is undertaken according to a Research Collaboration Agreement or Material Transfer Agreement entered into by the Tree of Life collaborator, Genome Research Limited (operating as the Wellcome Sanger Institute) and in some circumstances other Tree of Life collaborators.

## Data Availability

European Nucleotide Archive: *Vipera ursinii rakosiensis* (Hungarian meadow viper). Accession number PRJEB55895; https://identifiers.org/ena.embl/PRJEB55895 ( Wellcome Sanger Institute, 2023). The genome sequence is released openly for reuse. The *Vipera ursinii rakosiensis* genome sequencing initiative is part of the European Reference Genome Atlas Pilot Project ( https://www.erga-biodiversity.eu/pilot-project). All raw sequence data and the assembly have been deposited in INSDC databases. The genome will be annotated using available RNA-Seq data and presented through the Ensembl pipeline at the European Bioinformatics Institute. Raw data and assembly accession identifiers are reported in Table 1.

## Author information

Members of the Wellcome Sanger Institute Tree of Life Management, Samples and Laboratory team are listed here: https://doi.org/10.5281/zenodo.10066175.

Members of Wellcome Sanger Institute Scientific Operations: Sequencing Operations are listed here: https://doi.org/10.5281/zenodo.10043364.

Members of the Wellcome Sanger Institute Tree of Life Core Informatics team are listed here: https://doi.org/10.5281/zenodo.10066637.

Members of the Tree of Life Core Informatics collective are listed here: https://doi.org/10.5281/zenodo.5013541.
