# Supplementary material for: The genome sequence of the Hungarian meadow viper, Vipera ursinii rakosiensis (Méhely, 1893)
Source: Wellcome Open Res. 2025 Jan 15;9:404. Originally published 2024 Jul 26. [Version 2] doi: 10.12688/wellcomeopenres.22694.2 (PMC11427866; doi:10.12688/wellcomeopenres.22694.2)
Supplement: Peer review history

# Reviewer report: Reviewer response for version 2

**DOI:** 10.21956/wellcomeopenres.26052.r117757

**Published:** 2025-02-15

**Authors:** Drew Schield

**Affiliations:** 1 University of Virginia, Charlottesville, VA, USA

The authors have generated a high-quality reference genome for *Vipera ursinii*, which will be a valuable resource for research at various scales, from focused conservation genetics work on the species to broad comparative genomics investigations.

 The authors have also taken into account constructive feedback from previous reviewers, which has improved the presentation of the results and summary of the reference genome assembly.

 I have no additional concerns about this version of assembly report.

Are sufficient details of methods and materials provided to allow replication by others?

Yes

Is the rationale for creating the dataset(s) clearly described?

Yes

Are the datasets clearly presented in a useable and accessible format?

Yes

Are the protocols appropriate and is the work technically sound?

Yes

Reviewer Expertise:

Genomics, population genetics, evolution

I confirm that I have read this submission and believe that I have an appropriate level of expertise to confirm that it is of an acceptable scientific standard.

# Reviewer report: Reviewer response for version 2

**DOI:** 10.21956/wellcomeopenres.26052.r117495

**Published:** 2025-01-31

**Authors:** Paul Doniol-Valcroze

**Affiliations:** 1 CNRS, Univ. Montpellier, Montpellier, France

The authors present a chromosome level assembly for the highly endangered *rakosiensis *subspecies of the Meadow viper. Despite some very minor writing details outlined bellow, the rationale to produce such genomic resources is well justified and explained as well as the life history of the species or the origin of the sampled individual/population. I'll be very happy to see this genome note published and this genome made available for future studies.

**Background:**

- Rewrite the sentence about the ssp desciption: e.g. "was originally described [...] from types specimens originating from [...]"
- Ref for limited sexual dimorphism (end of 2nd paragraph)
- Change the sentence "Meadows and pastures [...] a mosaic of wet and dry grass habitats are favoured"
- I would deleted the mention to *small-sized lizards *when the authors explain that the species is insectivorous especially because they describe the opportunistic lizard consumption in the subsequent sentence: "The Hungarian meadow viper is mainly insectivorous, consuming grasshoppers, crickets and small-sized lizards. Adults opportunistically predate on vertebrates such as lizards and rodents."
- "The available [...] using next generation sequencing methods". Could be worth mentioning here the different and sometimes non concordant phylogenetic studies of the genus, a field which will certainly benefit from such genetic resources.

**Genome sequence report: **NA

**Methods:**

- Even is it is implicit here it could be worth mentioning that the specimen is a wild (not born in captivity) animal : "The female is a founder of the ex-situ captive population in the Hungarian Meadow Viper Conservation Centre."

Are sufficient details of methods and materials provided to allow replication by others?

Yes

Is the rationale for creating the dataset(s) clearly described?

Yes

Are the datasets clearly presented in a useable and accessible format?

Yes

Are the protocols appropriate and is the work technically sound?

Yes

Reviewer Expertise:

My work is interested in describing and understanding various mechanisms at the origin of biodiversity especially speciation, hybridization and migration. I use various approaches such as population genomics, phylogenomics, chemical ecology, deployment of multisensor-loggers or phenotyping. My main study models are Songbirds, Reptiles & Amphibians and Butterflies.

I confirm that I have read this submission and believe that I have an appropriate level of expertise to confirm that it is of an acceptable scientific standard.

# Reviewer report: Reviewer response for version 2

**DOI:** 10.21956/wellcomeopenres.26052.r117491

**Published:** 2025-01-30

**Authors:** Divya Tej Sowpati

**Affiliations:** 1 CSIR-Centre for Cellular and Molecular Biology, Hyderabad, India

I commend the authors for producing a high quality genome assembly of the Hungarian meadow viper. The article is complete in terms of understanding the genome quality.

**It would be nice to have the following aspects covered:**

 1. **Repeat content:** Distribution of interspersed as well as tandem repeats.

 2. Any analysis that was done on DNA methylation using the PacBio data.

Are sufficient details of methods and materials provided to allow replication by others?

Yes

Is the rationale for creating the dataset(s) clearly described?

Yes

Are the datasets clearly presented in a useable and accessible format?

Yes

Are the protocols appropriate and is the work technically sound?

Yes

Reviewer Expertise:

Computational genomics

I confirm that I have read this submission and believe that I have an appropriate level of expertise to confirm that it is of an acceptable scientific standard.

# Reviewer report: Reviewer response for version 2

**DOI:** 10.21956/wellcomeopenres.26052.r117753

**Published:** 2025-01-30

**Authors:** Alastair J. Ludington

**Affiliations:** 1 The University of Adelaide, Adelaide, Australia

**Article summary:**

 The Data Note submitted by Halpern et al. presents the fist high quality genome assembly of the endangered Hungarian meadow viper ( *Vipera ursinii rakosiensis*). This genome assembly, along with complementary sequencing data, will serve as a valuable resource for future ecological, conservation and demographic studies on *V. ursinii*, as well as for broader ecological and evolutionary research in herpetology. Using a combination of PacBio HiFi and Hi-C sequencing, the authors have assembled a 1.63Gb reference genome, consisting of 19 pseudomolecules (17 autosomes and the W and Z sex chromosomes), in addition to the mitochondrial genome. Assembly summary metrics, including BUSCO gene completeness (92.6%), Merqury K-mer completeness (99.09%), consensus quality (57.3) and assembly contiguity (N50 = 212.8Mb), indicate that the final assembly accurately represents the underlying data and is of high quality.

**Constructive comments:**

 The assembly metrics presented in the manuscript indicate that the reference genome is of high quality. A nice addition to the text could be a comment on how the quality of this genome assembly compares to other available reference genomes for snakes in the Viperidae family, or even more broadly. Specifically relating to chromosome number, sequence accuracy and relative levels of gene completeness.

 Second, there is mention of RNA-sequencing in the methods, though there is no mention of its use in the results. My assumption is that this is to be used for the annotation of protein coding genes, which if so, would be a valuable resource if provided in the future.

Are sufficient details of methods and materials provided to allow replication by others?

Yes

Is the rationale for creating the dataset(s) clearly described?

Yes

Are the datasets clearly presented in a useable and accessible format?

Yes

Are the protocols appropriate and is the work technically sound?

Yes

Reviewer Expertise:

Evolutionary Biology, comparative genomics

I confirm that I have read this submission and believe that I have an appropriate level of expertise to confirm that it is of an acceptable scientific standard.

# Reviewer report: Reviewer response for version 1

**DOI:** 10.21956/wellcomeopenres.24995.r95430

**Published:** 2024-09-26

**Authors:** Jia-Tang Li

**Affiliations:** 1 University of Chinese Academy of Sciences, Beijing, People's Republic of China, China

**Peer Review Report**

**Summary of the Article**

 This study presents the genome assembly of the Hungarian meadow viper ( *Vipera ursinii rakosiensis* ), a highly endangered snake species in Europe. The significance of this work lies in its potential contributions to conservation biology and serpentine genomics research. The authors have employed a combination of PacBio HiFi and Illumina sequencing technologies, enhanced by Hi-C, to successfully assemble a 1.63 Gb genome encompassing 19 chromosomal pseudomolecules, including the sex chromosomes and mitochondrial genome. The genome's integrity and quality have been highly appraised by BUSCO and Merqury metrics, indicating an exceptional assembly quality, which is vital for further genomic studies. 

**Evaluation Report**

 1. Rationale for Creating the Dataset

 The article articulates a well-defined rationale for the dataset creation, including the species' endangered status, biological significance, and implications for conservation efforts. The detailed context of sample collection also solidifies the justification for the dataset.

 2. Appropriateness of Protocols and Technical Soundness of the Work

 The study employs cutting-edge sequencing technologies and assembly strategies, which are recognized as state-of-the-art in genomics research.

 3. Sufficiency of Details on Methods and Materials

 The article provides a comprehensive experimental workflow, including sample processing, DNA and RNA extraction, sequencing platform selection, assembly strategies, and quality control measures, offering ample information for other researchers to replicate the study's methods.

 4. Presentation and Accessibility of the Dataset

 The dataset is made available through public databases with detailed access links and identifiers. The data is presented in standardized formats that are easy to utilize and analyze.

**Constructive Recommendations**

 1. While the genome assembly is commendable, it is recommended that the authors situate their findings within the broader context of serpentine genomics. By benchmarking against other assemblies, the authors can underscore the precision and accuracy of their work, providing additional layers of validation and confidence in the data presented.

 2. Genome annotation is a critical step in understanding the functions of genes and proteins, which is vital for revealing the biological characteristics and evolutionary relationships of species. It is hoped that the authors will promptly complete the genome annotation work and make the results publicly available.

Are sufficient details of methods and materials provided to allow replication by others?

Yes

Is the rationale for creating the dataset(s) clearly described?

Yes

Are the datasets clearly presented in a useable and accessible format?

Yes

Are the protocols appropriate and is the work technically sound?

Yes

Reviewer Expertise:

Evolutionary Biology, Functional Genomics

I confirm that I have read this submission and believe that I have an appropriate level of expertise to confirm that it is of an acceptable scientific standard.

# Reviewer report: Reviewer response for version 1

**DOI:** 10.21956/wellcomeopenres.24995.r95435

**Published:** 2024-09-18

**Authors:** Turk Rhen

**Affiliations:** 1 Department of Biology, University of North Dakota, Grand Forks, North Dakota, USA

Overall, this Data Note is well written and provides the first high-quality genome assembly for an endangered snake, the Hungarian meadow viper ( *Vipera ursinii rakosiensis*). This genome will be a valuable resource for current and future studies aimed at conservation and recovery of the species. The methods for DNA and RNA extraction, sequencing, and assembly of the genome are up to date and appropriate. The finished genome of 1.63 Gb has been assembled into 19 chromosomal pseudomolecules, including the sex chromosomes and the mitochondrial genome. Basic descriptive statistics indicate the genome assembly is of good quality: the assembly had a contig N50 of 2.1 Mb and a scaffold N50 of 213 Mb, *k*-mer completeness of 99.99%, and a BUSCO v5.3.2 completeness of 92.6%. The raw data and assembly are available in the link to the project accession in the European Nucleotide Archive.

**I have a few minor comments for the authors:**

- First, RNA-Seq on blood was done and the raw sequence data has been made available. However, the authors do not provide the RNA integrity number (RIN) of the sample that was sequenced. This is an important basic metric that should be reported to allow readers to judge whether the RNA sequence data is likely to be excellent, good, or poor.
- Second, the contrast and color scheme in Figure 5 showing the Hi-C contact map does not facilitate viewing of the chromosomes. The authors should consider revising this figure. Third, while I understand that Data Notes do not normally include any analyses or conclusions, it would be useful to provide some basic information about the karyotype of this species in the background section (to know if all chromosomes have been assembled). Similarly, a description of the range of assembled genome sizes in other snakes would help readers place this genome assembly in context.

Are sufficient details of methods and materials provided to allow replication by others?

Yes

Is the rationale for creating the dataset(s) clearly described?

Yes

Are the datasets clearly presented in a useable and accessible format?

Yes

Are the protocols appropriate and is the work technically sound?

Yes

Reviewer Expertise:

Genomics, sex determination, sexual differentiation, genetics, development

I confirm that I have read this submission and believe that I have an appropriate level of expertise to confirm that it is of an acceptable scientific standard.
